# Heading toward the Right Direction—Solution Package for Endoscopic Submucosal Tunneling Resection in the Stomach

**DOI:** 10.1371/journal.pone.0119870

**Published:** 2015-03-23

**Authors:** Jiaoyang Lu, Taotao Jiao, Yanmei Li, Ying Liu, Yanan Wang, Yatian Wang, Minhua Zheng, Xuefeng Lu

**Affiliations:** 1 Department of Gastroenterology, Qilu Hospital, Shandong University School of Medicine, Jinan, Shandong, China; 2 Department of Statistics, Shandong Provincial Hospital, Jinan, Shandong, China; 3 Department of General Surgery, Ruijin Hospital, Shanghai Jiao Tong University School of Medicine, Shanghai, China; National Cancer Center, JAPAN

## Abstract

**Background:**

The emerging submucosal tunneling and endoscopic resection (STER) technique provides definitive histological diagnosis as well as a therapeutic method for the gastric submucosal tumors (SMTs). We aim to present our experience and discuss key technical issues of STER.

**Methods:**

45 patients with gastric SMTs arising from MP received STER. First, a mucosal incision was made 3cm proximal to the tumour, a submucosal tunnel was subsequently built from the incision to the tumor. The tumor was gradually exposed and dissected from surrounding tissue and retrieved from the tunnel. The initial mucosal incision was closed by metal clips. For SMTs in the gastric fundus near cardia, the submucosal tunnel was built from lower esophagus, through the angle of His, to the tumor for resection.

**Results:**

STER was successfully performed in 43 patients; the other two were converted to surgery. Mean operating time was 79.3min (range 45–150min). Mean tumor size was 1.4cm (range 0.5–5cm). Of the total 47 resected SMTs, 36 were GISTs, 10 were leiomyomas and 1 was schwannoma. Complete resection was achieved in all patients. Intra-procedural peumoperitoneum occurred in 3 cases because of iatrogenic perforation, no special treatment was given. 7 patients presented with mild abdominal pain/distention and fever were given antibotics. No severe post-operative complication happened. No tumor recurrence occurred in the median 11 month follow-up period.

**Conclusion:**

Based on short-term follow-up observation, STER is a feasible, safe and minimally invasive method for the diagnosis and treatment of small (<3cm) SMTs in gastric body, antrum and proximal cardia.

## Introduction

Gastric submucosal tumors (SMTs) arising from the muscularis propria are mostly composed of gastrointestinal stromal tumors (GISTs) and leiomyomas. Small (<3cm), incidental SMTs are increasingly detected during endoscopic screening. The management of such tumors poses particular challenges to both gastroenterologists and surgeons due to various reasons, including the potential malignancy and unclear prognosis of GISTs, the suboptimal differential diagnostic yield of GIST from other, often benign SMTs, by endoscopic ultrasound guided fine-needle aspiration (EUS-FNA)[1–3], the cost-effectiveness of a major surgical intervention for a possible benign lesion [4,5] and the currently unavailable evidence-based surveillance policy.

Laparoscopic wedge resection for SMTs has been established, yet certain limitations exist. Extragastric wedge resection (EWR) was applied to tumors located in anterior and lateral wall but cannot be easily performed near gastric inlet or outlet due to possible post-operative stenosis or deformity; transgastric wedge resection (TWR) following anterior gastrostomy was applied to SMTs in posterior wall, yet this procedure is associated with additional risk of bleeding and intraperitoneal contamination from gastric juice leakage.

In recent years, endoscopic resection of small gastric SMTs is gaining popularity. This procedure provides definitive histological diagnosis as well as a minimally invasive therapeutic approach to such tumors. Traditionally, endoscopic submucosal excavation was applied [6–10], yet several technical limitations present, including high risk of perforation during excavation, poor endoscopic vision leading to difficulties in tumor exposure and hemostasis, and difficulties in closing the large, deep wound after tumor retrieval.

Recently, the submucosal tunneling and endosopic resection (STER) technique was developed to resect esophageal SMTs [11–14]. This recently developed technique, inspired by peroral endoscopic myotomy (POEM), features in dissecting a submucosal tunnel as an operating space for tumor resection, hence the mucosa covering is well preserved as a barrier against potential post-operative air and liquid leakage. Despite these technical advantages, STER is mainly performed in esophagus, whose tubular shape facilitates tunneling, but rarely in the more complex-shaped stomach [14]. In fact, in addition to adept endoscope maneuvering, operators need to dissect a tunnel from different directions to resect SMTs at different sites of stomach. Here, we report our experience of performing STER in stomach.

## Patients and Methods

We retrospectively analyzed information on 45 patients with gastric SMTs arising from the muscularis propria resected by STER since January, 2012. Tumor size and layer of origination were identified by endoscopic ultrasound (EUS) pre-operation; general indications for endoscopic resection included tumor size<3cm in diameter with no extra-luminal involvement; tumors located in the lesser curvature of gastric body/antrum, as well as in distal part of fundus were not resectable by STER due to retroflexion of endoscope. This study was approved by the institutional review board of Qilu hospital. Written informed consent was obtained from each patient.

### Equipments for gastric STER

A single channel endoscope (EPK-i7000; Pentax Optical Co. Ltd., Tokyo, Japan) attached with a transparent cap (MH-588; Olympus) was used during operation. A Dual knife (KD-650L; Olympus Optical Co. Ltd., Tokyo, Japan) was used for mucosal incision, submucosal tunneling and dissection of tumor (endocut mode 50w). A hot biopsy forceps (FD-410LR; Olympus) was used for hemostasis. In the final step, mucosal incision was closed by hemoclips (HX-610-135L Olympus). Other equipments included a high-frequency generator (300d; ERBE Elektromedizin GmbH, Tuebingen, Germany), and an argon plasma coagulation unit (APC300; ERBE Elektromedizin GmbH, Tuebingen, Germany). CO2 insufflation was used in all cases except for one patient who was comorbid with moderate chronic obstructive pulmonary disease (COPD).

### Procedures of gastric STER

Patients were placed in supine position with their right shoulder elevated by a pillow. All endoscopic procedures were carried out under general anesthesia with endotracheal intubation.

**Fig 1:**
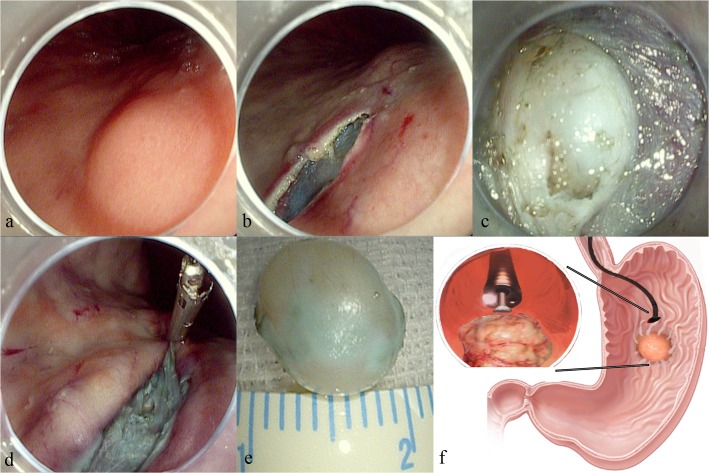
Submucosal tunneling and endosopic resection of a gastric submucosal tumor located in the posterior wall of gastric body. a: A gastric submucosal tumor located in the posterior wall of gastric body. b: A longitudinal mucosal incision was made about 3cm from targeted tumor after submucosal injection (the submucosal layer was stained in blue by indigo carmine). c: The tumor was gradually exposed by endoscopic dissection in the submucosal tunnel. d: After tumor resection and retrieval, the submucosal tunnel was closed by metal clips. e: The resected submucosal tumor in a diameter of 1cm. f: Schematic picture of the procedure; upper left picture showing the endoscope approaching the tumor in the tunnel for dissection.

**Fig 2:**
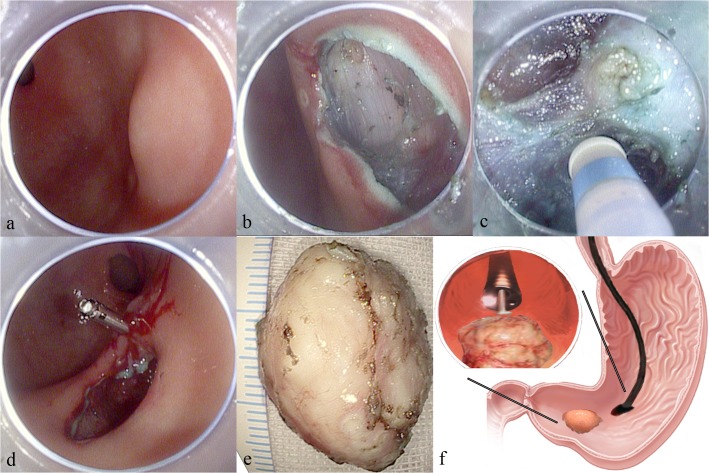
Submucosal tunneling and endosopic resection of a gastric submucosal tumor located in the antrum. a: A gastric submucosal tumor located in the antrum. b: A longitudinal mucosal incision was made about 3cm from targeted tumor after submucosal injection (the submucosal layer was stained in blue by indigo carmine). c: The tumor was gradually exposed by endoscopic dissection in the submucosal tunnel. d: After tumor resection and retrieval, the submucosal tunnel was closed by metal clips. e: The resected submucosal tumor in a maximum diameter of 3cm. f: Schematic picture of the procedure; upper left picture showing the endoscope approaching the tumor in the tunnel for dissection.

For SMTs located in the front/posterior side of gastric body/antrum, or in the great curvature, submucosal tunnel can be dissected in a direct view (Figs. 1 and 2). Procedures began by submucosal injection (5ml mixed solution of normal saline, indigo carmine (0.3%) and epinephrine (1:10000)) and subsequent mucosal incision around 3cm proximal to the targeted tumor (Figs. 1A, 1B, 2A and 2B). Endoscope then advanced in to dissect a submucosal tunnel all the way down to the site 2cm distal to the tumor to secure enough operating space. The tumor was then enucleated by dissection of surrounding submucosal tissue and smooth muscle fibers; full-thickness resection can be performed if the tumor was tightly attached to serosa or in an exophytic growth pattern (Figs. 1C and 2C). Meanwhile, pneumoperitoneum might happen; this was usually not serious as CO2 insufflation was used; nevertheless, paracentesis was necessary if heart rate/blood pressure instability occurred. Finally, after retrieval of tumor, visible bleeding in the MP defect was coagulated by argon plasma coagulation (APC) or electric biopsy forceps (Figs. 1D and 2D); the tunnel was lavaged repeatedly with normal saline if serosa was intact. The initial mucosal incision was closed by 4–6 metal clips in the end.

**Fig 3:**
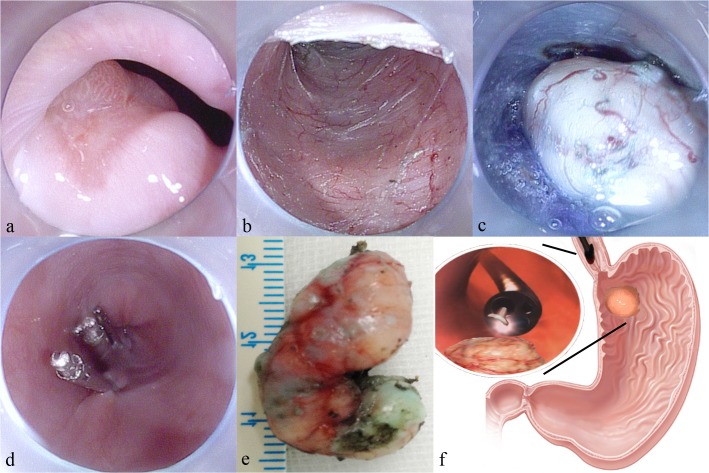
Endoscopic trans-cardiac submucosal tunneling dissection of a gastric submucosal tumor located in the fundus. a: A gastric submucosal tumor located in the fundus. b: A longitudinal mucosal incision was made in lower esophagus about 5cm from targeted tumor and a submucosal tunnel was built all the way down to the gastric fundal tumor. c: The tumor was gradually exposed by endoscopic dissection in the submucosal tunnel. d: After tumor resection and retrieval, the submucosal tunnel was closed by metal clips. e: The resected semi-ring-like submucosal tumor. f: Schematic picture of the procedure showing the endoscope approaching the tumor in the submucosal tunnel from lower esophagus, through the sharp angle of His, to the submucosal tumor for resection.

For SMTs located in the gastric fundus near cardia, tunneling by a retroflexed endoscope was impossible. Therefore, we developed a trans-cardiac tunneling technique to resect tumors in this area (Fig. 3) [15]. The mucosal incision was made in lower esophagus or cardia about 5cm proximal to the targeted SMT in fundus (Fig. 3A and 3B). The submucosal tunnel was dissected from esophagus, through cardia and finally reached the tumor for resection (Fig. 3C). Note that the submucosal space of the gastric fundus is hypervascularized, therefore careful prophylactic hemostasis by electric heating forceps is necessary; small hematomas happened occasionally, no special treatment was given. Small perforation (<1cm) of mucosa covering could be closed by clips after tumor resection (Fig. 3D).

### Histological examination

Tissue specimens were fixed in formalin and sectioned for pathological examination. Histological evaluation included cell type identification, overall cellularity, nuclear atypia, and calculation of mitotic index. Immunohistochemical staining for CD 117, CD 34, smooth muscle actin (SMA), and S-100 markers were used to identify tumor subtype. GISTs were classified into very low risk, low risk, moderate risk and high risk according to size and mitotic count [16]. Complete resection (R0 resection) was defined as resection with intact capsule and tumor-free margin.

### Postoperative management and follow-up

Vital signs of patients including heart rate, blood pressure and oxygen saturation were monitored to stabilization. A gastric decompression tube was placed for acid drainage and for detection of post-operative hemorrhage. Haemostatic agent (Etamsylate) and proton pump inhibitor (PPI) were given intravenously for normally 3 days before recovery to full liquid diet; oral PPI was then prescribed for another 4 days. Prophylactic antibiotics were prescribed for only one day post operation if no fever occurred. Potential post-operative symptoms included fever and abdominal pain/distention. Abdominal X-ray or CT could be used to confirm free-air leakage if suspected. Severe peumoperitoneum could be relieved by paracentesis by a 20 gauge needle. Patients were usually discharged several days after the procedure and were followed up by standard endoscopy to check incision healing and tumor recurrence in 2 and 6 months post operation, then annually thereafter (patients with leiomyoma or very-low risk GISTs were not required for follow-up if R0 resection was achieved).

## Results

STER was successfully performed in 43 out of 45 patients. Two patients were transferred to laparoscopic surgery, one with a 3cm difficult-to-resect exophytic tumor and the other patient presented consistent bleeding during operation (Note both cases were in our early phase of learning curve).

The mean age of the 43 patients (with 47 tumors) was 54.2y (range 35–75y); The mean operating time was 79.3min (range 45–150min). The mean tumor size was 1.4cm (range 0.5–5cm). 18 tumors located in fundus near cardia were resected by trans-cardiac submucosal tunneling technique; other tumors were all resected in regular gastric submucosal tunnels. Under pathologic examination, 36 were GISTs, 10 were leiomyomas and 1 was schwannoma. All GISTs were categorized into very low or low risk of malignancy. Complete resection (R0 resection) was achieved in all 43 patients.

During operation, small mucosal lacerations (<1cm) occurred in 4 patients and were closed by metal clips after tumor resection. Iatrogenic perforation was required in 3 cases, and intra-procedural peumoperitoneum occurred, no special treatment was given. 7 patients presented with mild abdominal pain/distention with body temperature above 38C; free air was detected by CT in 4 of these patients and intravenous antibiotics were given; symptoms of all 7 patients relieved in 3days. No patients developed GI leakage, delayed bleeding or secondary infection. No tumor recurrence happened in the median 11 month follow-up period.

## Discussion

Endoscopic resection provides a solution for the clinical dilemma of small gastric SMTs. Compared to open surgery or laparoscopic wedge resection which keeps sufficient surgical margin (>2cm), endoscopic methods only dissect along the margin of SMTs to keep the integrity of tumor capsule, and an en-bloc resection with negative margin of tumor cells under histologic examination is considered as a complete resection (R0 resection). The implementation of this procedure is based on the observation that small gastric SMTs are always encapsulated by a fibrous capsule and grow in a non-invasive pattern, with no lymph node metastasis [17]. Oncologic outcomes after endoscopic resection is encouraging: no tumor recurrence occurred during follow-up period up to 44 month in published literatures [8,9,18], although long-term follow up is still warranted.

Several differences lie between STER in stomach and the established STER in esophagus. In esophagus, mucosal incision is often made 5cm proximal to the lesion, whereas in stomach, 3cm is enough due to elasticity of gastric wall. Subsequently, because of this elasticity and the relatively large gastric cavity, tunneling may deviate from the right direction, leading to failure of detecting targeted tumor in the tunnel, therefore constantly adjusting the route of tunneling is necessary. This “getting lost” in the tunnel may as well be prevented by tunneling close to the MP layer instead of to the mucosal layer. During tumor enucleation, if full thickness resection was required, special attention should be paid to prevent the tumor from falling into the peritoneal cavity because stomach moves relative freely in the peritoneal space. In addition, stomach is more hypervascularized than esophagus, hence prompt hemostasis by electric heating forceps is required.

The management of gastric fundus tumors near cardia has been a tough problem as the risk of gastric inlet deformity or stenosis was high after laparoscopic wedge resection [19,20]. In this report, we also describe a trans-cardiac submucosal tunneling technique for the resection of such tumors. This procedure, as described above, is performed in a direct view, avoiding retroflexion of endoscope. In addition, the mucosal incision made in esophagus was away from gastric juice erosion. During the procedure, the location of mucosal access in lower esophagus is depend on the tumor location in the fundus with respect to quadrant and its distance from cardia. Generally, the distance from mucosal access to fundus tumor is about 5cm. Subsequently, during submucosal tunneling, the esophago-gastric junction can be identified by anatomical landmarks such as the ending of palisade vessels and the hypervascularized gastric submucosal layer.

**Fig 4:**
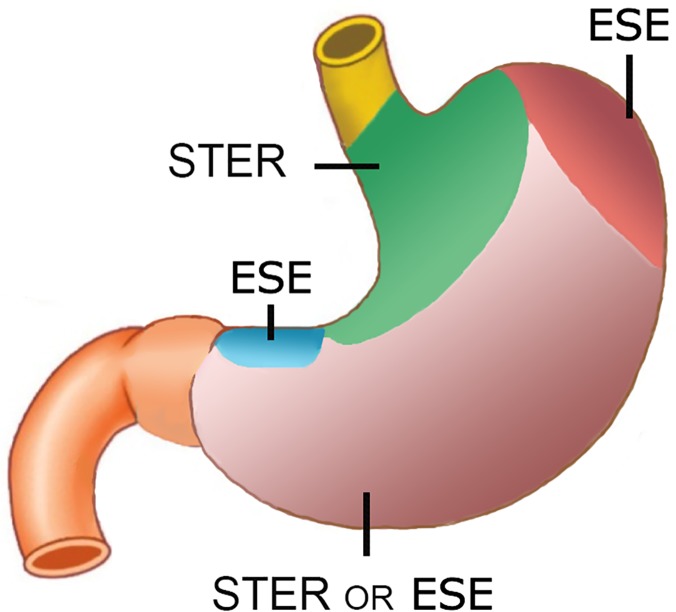
Different endoscopic resection techniques should be adopted for gastric submucosal tumors in different area. For tumors located in the circular area around cardia, although ESE can be performed in a retroflexed fashion, we still recommend transcardiac submucosal tunneling technique in a direct view. For tumors in distal fundus or lesser curvature, ESE in a retroflexed fashion is the only choice. For tumors located in most areas of gastric body, both ESE and STER are suitable.

Comparison between STER and endoscopic submucosal excavation (ESE) is helpful to find strengths and weaknesses of both techniques. ESE, as a technical extension of endoscopic submucosal dissection (ESD), is currently the main choice for endoscopic resection of gastric SMTs. This direct excavation technique can be used to resect SMTs in all parts of stomach, although been performed in a retroflexed fashion in lesser curvature or fundus is challenging. However, the lesser curvature is still a tabooed area for gastric STER. While the trans-cardiac tunneling technique, comfined by the maximum length of submucosal tunnel, can reach fundus SMTs as far as 8cm below cardia; fundus SMTs not within this area should still be resected by ESE. In short, we would like to recommend different endoscopic procedures for the resection of gastric SMTs in different location (**Fig. 4**).

In areas which STER and ESE are both applicable, STER is a preferred choice. The mucosal incision in STER is clean, neat and easy to close, while incision after ESE is often large, irregular, swelled and closed in high tension. Note that some operators make circumferential incision (the covering mucosa is removed) during ESE to get better vision and ease operation, this method will leave a large, deep gastric wall defect after operation; the gastric wall defect will delay wound healing, increase risk of air/liquid leakage and potential peritoneal infection. When a full-thickness resection is required, the large gastric wall defect is often difficult to close. Metal clips can only approximate the mucosal layer; other full-thickness closure devices are currently used in animal experiment. Therefore, STER has significant advantage in resecting SMTs in deep MP/ tightly attached to serosa.

In sum, based on short-term follow up observation, STER is a safe and minimally invasive way to resect gastric SMTs arising from muscularis propria. Long-term follow-up data is still required as GIST is a slow growing sracosa and low-risk GISTs tend to relapse later than high risk ones. In addition, STER remains as a challenging technique which requires intensive training; endoscopists need to start from easy procedures, such as STER in the esophagus and the antrum, before advancing to difficult areas in the stomach.
